# Supplementary material for: Modeling glioblastoma heterogeneity as a dynamic network of cell states
Source: Mol Syst Biol. 2021 Sep 16;17(9):e10105. doi: 10.15252/msb.202010105 (PMC8444284; doi:10.15252/msb.202010105)
Supplement: Supplementary file 6 — Source Data for Figure 5 [file MSB-17-e10105-s004.zip › Figure5A_sourcedata/GSEA_3071/nosigdb_stateV.GseaPreranked.1622795159954/ANASTASSIOU_MULTICANCER_INVASIVENESS_PMID_22208948.html]

Details for gene set ANASTASSIOU\_MULTICANCER\_INVASIVENESS\_PMID\_22208948[GSEA]

|  || Dataset | state43071 |
| Phenotype | NoPhenotypeAvailable |
| Upregulated in class | na\_neg |
| GeneSet | ANASTASSIOU\_MULTICANCER\_INVASIVENESS\_PMID\_22208948 |
| Enrichment Score (ES) | -0.7846085 |
| Normalized Enrichment Score (NES) | -3.5095239 |
| Nominal p-value | 0.0 |
| FDR q-value | 0.0 |
| FWER p-Value | 0.0 |
Table: GSEA Results Summary

  

Fig 1: Enrichment plot: ANASTASSIOU\_MULTICANCER\_INVASIVENESS\_PMID\_22208948      
 Profile of the Running ES Score & Positions of GeneSet Members on the Rank Ordered List

  

| PROBE | GENE SYMBOL | GENE\_TITLE | RANK IN GENE LIST | RANK METRIC SCORE | RUNNING ES | CORE ENRICHMENT || 1 | COL1A2 |  |  | 1028 | 0.614 | -0.1847 | No |
| 2 | LRRC15 |  |  | 1458 | 0.491 | -0.2576 | No |
| 3 | RAB31 |  |  | 1604 | 0.459 | -0.2771 | No |
| 4 | PLAU |  |  | 1863 | 0.408 | -0.3189 | No |
| 5 | ACTA2 |  |  | 2227 | 0.359 | -0.3816 | No |
| 6 | TMEM158 |  |  | 2340 | 0.343 | -0.3968 | No |
| 7 | PRRX1 |  |  | 2674 | 0.302 | -0.4548 | No |
| 8 | TIMP3 |  |  | 2826 | 0.286 | -0.4785 | No |
| 9 | MMP2 |  |  | 3234 | -0.257 | -0.5513 | No |
| 10 | RCN3 |  |  | 4033 | -0.396 | -0.6962 | No |
| 11 | ASPN |  |  | 4291 | -0.471 | -0.7367 | No |
| 12 | MFAP5 |  |  | 4343 | -0.485 | -0.7378 | No |
| 13 | EDNRA |  |  | 4361 | -0.489 | -0.7323 | No |
| 14 | VCAN |  |  | 4491 | -0.537 | -0.7473 | No |
| 15 | LOXL2 |  |  | 4624 | -0.605 | -0.7616 | No |
| 16 | NTM |  |  | 4695 | -0.643 | -0.7635 | No |
| 17 | TNFAIP6 |  |  | 4764 | -0.688 | -0.7642 | No |
| 18 | LGALS1 |  |  | 4784 | -0.699 | -0.7553 | No |
| 19 | THY1 |  |  | 4874 | -0.781 | -0.7583 | No |
| 20 | ITGBL1 |  |  | 5013 | -0.955 | -0.7676 | Yes |
| 21 | NUAK1 |  |  | 5025 | -0.983 | -0.7522 | Yes |
| 22 | COPZ2 |  |  | 5069 | -1.073 | -0.7412 | Yes |
| 23 | GLT8D2 |  |  | 5079 | -1.094 | -0.7235 | Yes |
| 24 | LOX |  |  | 5092 | -1.128 | -0.7057 | Yes |
| 25 | COL11A1 |  |  | 5110 | -1.178 | -0.6879 | Yes |
| 26 | COL5A2 |  |  | 5112 | -1.189 | -0.6669 | Yes |
| 27 | SERPINF1 |  |  | 5158 | -1.351 | -0.6514 | Yes |
| 28 | BGN |  |  | 5216 | -1.654 | -0.6328 | Yes |
| 29 | DCN |  |  | 5233 | -1.798 | -0.6038 | Yes |
| 30 | SNAI2 |  |  | 5241 | -1.903 | -0.5713 | Yes |
| 31 | CDH11 |  |  | 5245 | -1.943 | -0.5372 | Yes |
| 32 | PCOLCE |  |  | 5248 | -1.999 | -0.5020 | Yes |
| 33 | PDGFRB |  |  | 5253 | -2.045 | -0.4663 | Yes |
| 34 | FBN1 |  |  | 5263 | -2.234 | -0.4282 | Yes |
| 35 | COL6A2 |  |  | 5265 | -2.276 | -0.3879 | Yes |
| 36 | COL5A1 |  |  | 5274 | -2.455 | -0.3457 | Yes |
| 37 | LUM |  |  | 5277 | -2.507 | -0.3014 | Yes |
| 38 | INHBA |  |  | 5282 | -2.697 | -0.2541 | Yes |
| 39 | FN1 |  |  | 5287 | -2.866 | -0.2038 | Yes |
| 40 | COL3A1 |  |  | 5292 | -3.630 | -0.1399 | Yes |
| 41 | COL6A3 |  |  | 5293 | -3.722 | -0.0736 | Yes |
| 42 | COL1A1 |  |  | 5294 | -4.144 | 0.0002 | Yes |
Table: GSEA details [plain text format]

  

Fig 2: ANASTASSIOU\_MULTICANCER\_INVASIVENESS\_PMID\_22208948: Random ES distribution      
 Gene set null distribution of ES for **ANASTASSIOU\_MULTICANCER\_INVASIVENESS\_PMID\_22208948**
